# Crop diversity promotes the recovery of fungal communities in saline-alkali areas of the Western Songnen Plain

**DOI:** 10.3389/fmicb.2023.1091117

**Published:** 2023-02-01

**Authors:** Bin Li, Xiaoqian Liu, Dan Zhu, Heng Su, Kaiwen Guo, Guangyu Sun, Xin Li, Lei Sun

**Affiliations:** ^1^College of Resources and Environment, Northeast Agricultural University, Harbin, China; ^2^College of Life Science, Northeast Forestry University, Harbin, China; ^3^School of Forestry, Northeast Forestry University, Harbin, China

**Keywords:** soil fungal communities, saline-alkali soils, cropping patterns, MiSeq sequencing, co-occurrence network, community assembly

## Abstract

**Introduction:**

Phytoremediation is an effective strategy for saline land restoration. In the Western Songnen Plain, northeast China, soil fungal community recovery for saline phytoremediation has not been well documented among different cropping patterns. In this study, we tested how rotation, mixture, and monoculture cropping patterns impact fungal communities in saline-alkali soils to assess the variability between cropping patterns.

**Methods:**

The fungal communities of the soils of the different cropping types were determined using Illumina Miseq sequencing.

**Results:**

Mixture and rotation promoted an increase in operational taxonomic unit (OTU) richness, and OTU richness in the mixture system decreased with increasing soil depth. A principal coordinate analysis (PCoA) showed that cropping patterns and soil depths influenced the structure of fungal communities, which may be due to the impact of soil chemistry. This was reflected by soil total nitrogen (TN) and electrical conductivity (EC) being the key factors driving OTU richness, while soil available potassium (AK) and total phosphorus (TP) were significantly correlated with the relative abundance of fungal dominant genus. The relative abundance of *Leptosphaerulina*, *Alternaria*, *Myrothecium*, *Gibberella*, and *Tetracladium* varied significantly between cropping patterns, and *Leptosphaerulina* was significantly associated with soil chemistry. Soil depth caused significant differences in the relative abundance of *Fusarium* in rotation and mixture soils, with *Fusarium* more commonly active at 0–15 cm deep soil. Null-model analysis revealed that the fungal community assembly of the mixture soils in 0–15 cm deep soil was dominated by deterministic processes, unlike the other two cropping patterns. Furthermore, fungal symbiotic networks were more complex in rotation and mixture than in monoculture soils, reflected in more nodes, more module hubs, and connectors. The fungal networks in rotation and mixture soils were more stable than in monoculture soils, and mixture networks were obviously more connected than rotations. FUNGuild showed that the relative proportion of saprotroph in rotation and mixture was significantly higher than that in monocultures. The highest proportion of pathotroph and symbiotroph was exhibited in rotation and mixture soils, respectively.

**Discussion:**

Overall, mixture is superior to crop rotation and monocultures in restoring fungal communities of the saline-alkali soils of the Western Songnen Plain, northeast China.

## Introduction

Soil salinization has become a global concern, threatening land use as well as food supply (Hajiboland, 2013; Chaganti et al., 2015; Sun et al., 2017; Xie et al., 2017). In China, saline-alkali soils have reportedly reached 3.67 × 10^7^ ha and are increasing at a rate of 1% per year (Li and Wang, 2018). Destruction of terrestrial ecological balance and deterioration of important environments is attributed to soil salinization, has led to the abandonment of saline-alkali farmland (Zhao et al., 2018). Soil degradation due to salinization and alkalization has become a global problem, and there is an urgent need to develop measures to improve the current situation.

Planting salt and alkali-resistant plants are excellent choice for the remediation of saline-alkali soils. It has been reported that phytoremediation strategies can be used to significantly increase microbial activity and restore soil fertility while decreasing the risk of saline-alkali soils (Boyrahmadi and Raiesi, 2018; Luo et al., 2018; Choudhary et al., 2022). Microorganisms are an essential player in enhancing saline-alkali soils (Yang and Sun, 2020; Liu et al., 2022). Phytoremediation can effectively slow secondary soil salinization by regulating the composition of the soil microbial community and promoting nutrient accumulation in crops (Cai et al., 2022). Several studies have been conducted on the response of soil fungal communities to the degree of soil salinization (Perez-Llano et al., 2020; Luo, J. Q. et al., 2021; Chen et al., 2022). For instance, it was noted that the Shannon and Chao indices of fungal decreased significantly with increasing salinization gradients in northern Shandong Province, China (Yang and Sun, 2020). Moreover, the fungal community structure and fungal composition differed under moderate and severe salt stress conditions (Liu et al., 2022). Nevertheless, information on the fungal communities present in the saline-alkali soils of different cropping patterns is still insufficient.

The variability of soil parameters and different cropping patterns greatly influences the distribution of soil microbial communities, and this should not be overlooked (Borrell et al., 2017; Granzow et al., 2017). The diversification of cropping systems has been suggested as a way to enhance soil microbiology to promote more sustainable and resilient agricultural systems (Altieri, 1999). Rotation and mixture are being promoted as ecologically sustainable agriculture (Verret et al., 2020; Xie F. C. et al., 2020; Xie Y. Y. et al., 2020; Jalli et al., 2021; Cui et al., 2022). It is well known that there are benefits to soil microbial communities and crop productivity in rotation and mixture (Fujiishi et al., 2020; Benitez et al., 2021; He et al., 2021; Che et al., 2022). For example, by monitoring the changes of soil microbial communities in seven-crop rotations and monoculture systems in greenhouse vegetable production in Harbin, China, He et al. (2021) found that fungal abundance was significantly increased in rotation systems. Crop rotations also improve soil nutrients by affecting soil microbial community composition (Zhang et al., 2019). In addition, the mixture promotes soil biomass through its contribution to soil organic carbon and enhances microbial processes and the diversity of decomposition metabolism (Chavarria et al., 2016). Highly diverse mixture resulted in a significant increase of 20% in the individual microbiome, including bacteria, actinomycetes, saprophytes, and mycorrhizal fungi (Simeone et al., 2020).

Fungi, which are abundant and fast-spreading, are ubiquitous, important microorganisms in soil ecosystems (Finlay, 2002). Fungi are sensitive to saline-alkali soils and are more susceptible to saline-alkali stress than bacteria (Gavrichkova et al., 2020; Hou et al., 2021). Fungi represent one of the most diverse groups of living organisms and play an essential role in terrestrial ecosystems, functioning as plant decomposers, pathogens, and symbionts (Zeilinger et al., 2016). Therefore, it is important to identify the driving forces of community assemblages to explain the mechanisms of species coexistence and maintenance (Rosindell et al., 2011; Ning et al., 2020). There are two different processes that explain the changes in microbial communities to address their relative importance in governing community assembly: deterministic (niche-based) and stochastic (neutral) processes (Zhou et al., 2017). Fungal communities are strongly impacted by processes such as dispersal, ecological drift and stochastic recruitment (Peay et al., 2010, 2012; Bahram et al., 2016). The relative contributions of these processes may vary depending on the type and nature of the ecosystem and understanding the relative contribution of each process in different environments remains challenging (Beck et al., 2015). A correlation-based network analysis has been widely used to infer microbial interactions (Faust and Raes, 2012; Duran et al., 2018; Xiong et al., 2018). Cooperative and competitive interactions among microbial species can influence community stability (Liu et al., 2020; Mundra et al., 2021). Heisey et al. (2022) found that the compost network (44 nodes and 31 edges) and urea network (18 nodes and 11 edges) were more complex than the no fertilizer control network (14 nodes and 7 edges) in fungal communities. A well-rounded understanding of the interactions between cropping systems, soil, and fungal communities in saline-alkali soil is therefore essential for the development of sustainable agroecosystems. However, few studies have been conducted on the soil fungal communities of different cropping patterns in saline-alkali soils, especially in the Western Songnen Plain, northeast China.

Therefore, in this study, we investigated the fungal communities in three different cropping systems, monoculture, rotation, and mixed cropping, in the Western Songnen Plain, northeast China and assessed differences in fungal communities by Illumina MiSeq sequencing methods. The objective of our research was to examine if various facets of the soil fungal community, like diversity and composition, were sensitive to changes in cropping systems and soil depths. We hypothesized that (1) complex restoration cropping systems would lead to increased soil fungal community diversity compared to monocultures; (2) the adoption of rotation and mixture will lead to variations in fungal community composition; (3) rotation and mixture affect the pattern of co-occurrence networks and community assembly.

## Materials and methods

### Study area

Sifang Mountain Farm is situated in Zhaodong City, Heilongjiang Province, China (E125° 45′–126° 30′, N46° 12′–46° 22′). It has flat terrain, an altitude ranging 135 and 145 m, and the landform belongs to the Songnen Plain. The soil is carbonate meadow alkaline soil and carbonate meadow soil. The average annual temperature is 2.4°C, the annual evaporation is 1,662 mm, the average annual rainfall is 396 mm, the maximum temperature is 39.0°C, the minimum temperature is −37.5°C, and the annual effective temperature is 2,500–2,700°C. In this area, the starting soil pH was as high as 11.00, and it was an alkali patch of bare ground without any vegetation.

### Experimental design and sample collection

**Figure 1:**
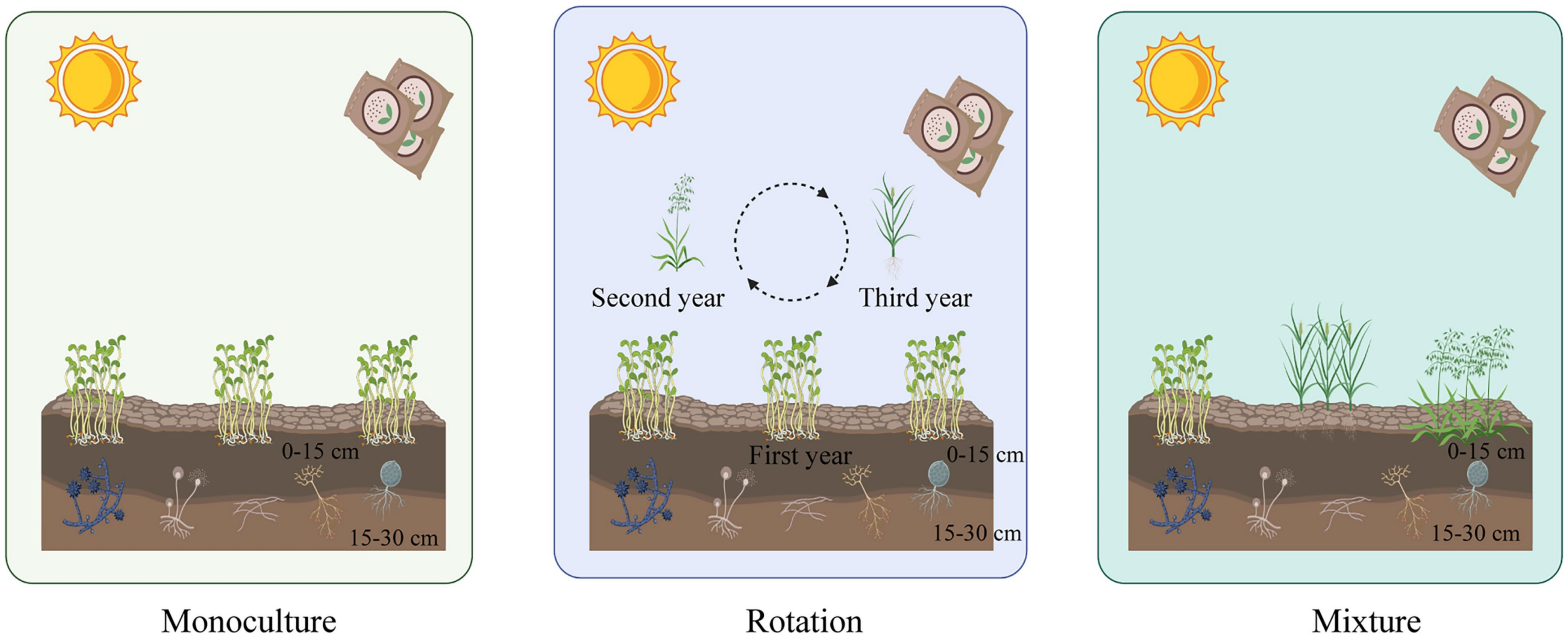
A diagrammatic sketch to show our experimental design. Three cropping patterns, monoculture, rotation, and mixture systems were cropped in each of the three plots. Alfalfa is cropped in monoculture soils yearly. A rotation of alfalfa–oat–tall wheatgrass that has been managed in this way for 13 years, with annual alfalfa in the first year, followed by oats in the second year and tall wheatgrass in the third year, in a three-year rotation. A mixture system plot where alfalfa, oats, and tall wheatgrass were grown simultaneously.

This study was carried out in three plots in an experimental agricultural field. These plots consisted of: (a) an alfalfa monoculture system, where alfalfa was grown yearly; (b) a rotation of alfalfa–oat–tall wheatgrass that has been managed in this way for 13 years, with annual alfalfa in the first year, followed by oats in the second year and tall wheatgrass in the third year, in a three-year rotation; and (c) a mixture system plot where alfalfa, oats, and tall wheatgrass were grown simultaneously, where each crop was planted tightly together, almost interchanging in every other row. All plots had similar soil and climatic conditions and were subjected to the same fertilization and field management during the experimental period (Figure 1).

Soil samples were collected on 17 August 2017 from three plots, which had all been planted to alfalfa. Samples were collected before the alfalfa harvest. Soil was sampled from soil 0–15 and 15–30 cm depth in each replicated plot using a soil auger (8-cm diameter and 15-cm deep). Five samples were collected following an “S” shape, and then thoroughly mixed to obtain one composite sample for each replication, resulting in a total of 18 plot-level samples. After removing materials, such as roots and debris, each composite field soil sample was homogenized, placed in sterile plastic bags, and immediately shipped to the laboratory. The soil was then passed through a 2 mm sieve and visible debris was removed. Every soil sample was separated into two parts. One part was stored at −80°C for DNA extraction and the other part was air dried at room temperature to test soil chemical properties.

### Determination of soil chemical properties

The soil pH and electrical conductivity (EC) were measured at a soil/water ratio of 1:5 (weight:volume). Soil organic matter content (SOM) was determined using the dichromate oxidation method with heating (K_2_Cr_2_O_7_-H_2_SO_4_). Soil total nitrogen (TN) content was determined using the Kjeldahl method. Soil total phosphorus (TP) and available phosphorus (AP) were extracted by HClO_4_-H_2_SO_4_ digestion and NaHCO_3_ methods, respectively. Soil available potassium (AK) was extracted with 1 M ammonium acetate. All soil chemistry was determined following the methods previously described by Bao (2000).

### DNA extraction and Illumina MiSeq sequencing

Soil microbial DNA was extracted from 0.5 g of fresh soil sample using a Power Soil DNA Isolation Kit (Mo Bio Laboratories, Inc., Carlsbad, CA, United States) according to the manufacturer’s instructions. The amount of DNA was measured using a NanoDrop 2000 spectrophotometer (Thermo Scientific, United States). The copy numbers of the fungal ITS gene were assayed using a ABI GeneAmp^®^ 9700 with the primers ITS1F (5′-CTTGGTCATTTAGAGGAAGTAA-3′) and ITS2R (5′-GCTGCGTTCT TCATCGATGC-3′) (Zhang et al., 2016). PCR reactions were performed in triplicate in a 20 μL mixture containing 2 μL of 10 × FastPfu Buffer, 2 μL of 2.5 mMdNTPs, 0.8 μL (5 μM) of each primer, 0.2 μL of TaKaRa rTaq Polymerase, 0.2 μL of BSA, 10 ng of template DNA, and ddH2O to make final volume of 20 μL. PCR products from all samples were mixed and detected using 2% agarose gel electrophoresis, and the PCR products were recovered by cutting the gel using an AxyPrep DNA Gel Recovery Kit (AXYGEN). PCR products were quantitatively detected using a QuantiFluor^™^-ST Blue fluorescence quantitative system (Promega). The MiSeq sequencing library was constructed using TruSeq^™^ DNA Sample Prep Kit, which was sequenced using the PE300 strategy. The PCR products were purified, pooled in equimolar concentrations, and paired-end sequenced (2 × 300) using an Illumina MiSeq platform by Annuoida Gene Technology Co., LTD., Beijing, China.

### Bioinformatics analysis

Low-quality raw data sequences (average base quality score < 20 and length < 250 bp) were eliminated using Trimmomatic and merged by FLASH (Fast Length Adjustment of Short reads) (Magoc and Salzberg, 2011; Bolger et al., 2014). In total, 1,119,035 high-quality fungal reads were obtained in this study. OTUs were generated at a ≥ 97% similarity level using the USEARCH v7.1 pipeline (Edgar, 2013). The remaining sequences were denoised (Schloss et al., 2009) and aligned against the UNITE v7.0 fungal ITS gene database using Mothur (Koljalg et al., 2013). The original sequences were deposited in the NCBI Sequence Read Archive (SRA) with the accession number PRJNA895952.

### Statistical analysis

The fungal microbial community diversity indices were calculated using the vegan package in R (Oksanen et al., 2010). According to Bray–Curtis dissimilarity matrices, a principal coordinates analysis (PCoA) and Mantel test were conducted using the vegan library in R. The correlations between diversity indices and soil properties were examined by Spearman linear regressions. A random forest analysis of soil properties and diversity was performed using the Random Forest package in R, respectively (Liaw and Wiener, 2002). The correlation between the dominant fungal genera in communities and soil properties was examined using the Spearman function in the Hmisc package in R (Harrell and Dupont, 2020).

To explore the fungal community assembly, we tested a null model using the method described by Stegen et al. (2013). The βNTI and Raup-Crick matrix (RC_bray_) were calculated using the picante and vegan packages in R, respectively (Kembel et al., 2010). In addition, we used fitted negative binomial generalized log-linear models from the edgeR package to examine the variation of OTU abundance and differential enrichment of OTUs among the three cropping patterns (Robinson et al., 2010). We focused only on OTUs that were abundant in the fungal community, so we filtered for OTUs in the top 50% of abundance. Spearman’s correlation coefficients were performed using the psych package in R (Revelle, 2013). *p*-values were calculated and corrected using the false discovery rate (FDR; Schmutter et al., 2016). Then, we used a coefficient < 0.7 and a *p*-value >0.01 as thresholds to filter out the OTUs with a low correlation before building the network (Widder et al., 2014). Using the igraph package in R, three cropping patterns and their topological properties, including connectivity, averaging, and modularity, were constructed and visualized (Csardi and Nepusz, 2006; Guan et al., 2020). Thresholding Zi and Pi were used to test the topological role of each node (Guimera and Amaral, 2005). Despite our small sample size (*n* = 3 for each treatment), we found that the effect of cropping patterns was much greater than soil depths in shaping fungal community structure (fungal community diversity and composition). We therefore combined all samples from two soil depths for each cropping pattern, allowing for six samples per cropping pattern for the network analysis. The functional profiles of fungi were predicted using the FUNGuild database (Nguyen et al., 2016).

The impact of cropping pattern and soil depth on community diversity was investigated by a two-way ANOVA and Kruskal–Wallis test. One-way ANOVA was used to test the impact of each treatment on the composition of the soil fungal community. Variations between treatments were then examined using the Duncan’s *post-hoc* test, *p* < 0.05.

## Results

### Fungal community richness and diversity

**Figure 2:**
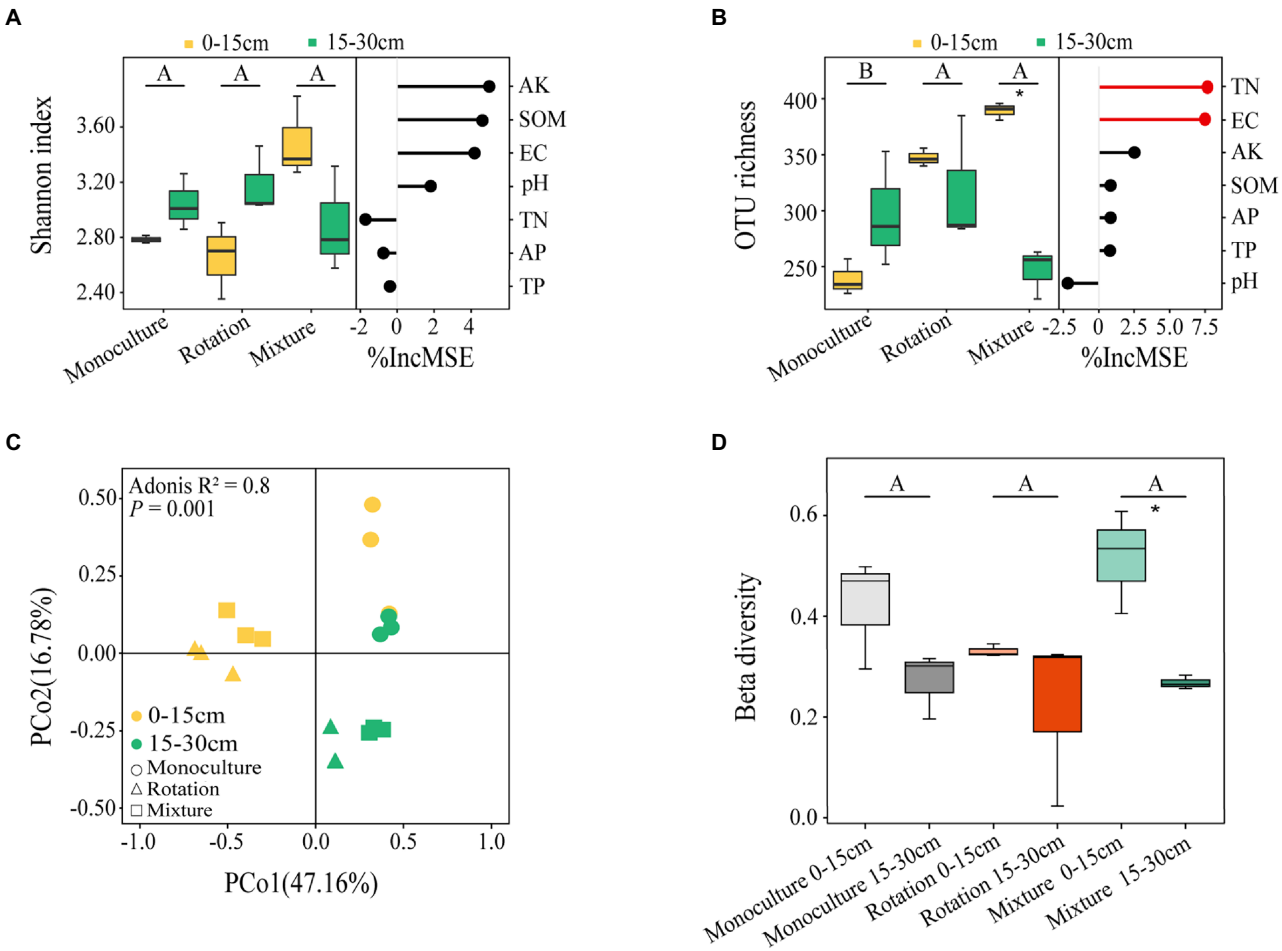
Shannon index **(A)** and OTU richness **(B)** of fungal communities at two soil depths for three cropping patterns and the importance of random forest prediction of soil variables on them. Based on Bary-Curtis distances, PCoA **(C)** and beta diversity index **(D)** of fungi community at the OTU level; Adonis tests for significant effects of different cropping patterns and soil depths on overall differences in β-diversity of microbial community structure and paired tests for differences between samples. Different letters indicate significant differences between groups (*p* < 0.05). A significant difference between soil depths and statistical significance is indicated as follows. *p ≤ 0.05.

A total of 1,119,035 high quality reads for fungal communities were obtained and clustered into 971 OTUs according to a 97% sequence similarity. The distribution of OTUs in the three different cropping patterns from soil collected from two depths is shown in Supplementary Figure 1. According to the two-way ANOVA, the OTU richness was significantly affected by cropping patterns and soil depths (Supplementary Table 2). The beta diversity index also was significantly affected by soil depth (Supplementary Table 2). The OTU richness of the rotation and mixture were significantly higher than that in monoculture fields (Figure 2B). The increase in soil depth caused a significant reduction in the OTU richness (*p* < 0.05) and beta diversity index (*p* < 0.05) of the mixture (Figures 2B,D). The OTU richness of mixture in 0–15 cm deep soil was 57.83% greater than that of in 15–30 cm deep soil (Figure 2B). The beta diversity index of mixture in the 0–15 cm soil layer was 92.59% greater than that in 15–30 cm soil layer (Figure 2D). The PCoA also indicated a significantly different fungal community structure in the two soil depths of the three cropping patterns, which was confirmed by Adonis’ test (Figure 2C).

Based on the random forest analysis, TN and EC were the main drivers of OTU richness (Figure 2B). In addition, the AK was significantly linearly associated with the Shannon index (Supplementary Figure 2A); the TN and EC were significantly linearly associated with the OTU richness (Supplementary Figure 2B). Similarly, the Mantel test also indicated that TP, AK, and EC were associated with fungi communities (Table 1).

**Table 1:** Mantel tests of the soil fungal community with soil variables.

Variables	Soil fungal community	
	*r*	*p*
pH	0.148	0.087
SOM	−0.029	0.516
TN	0.238	0.053
TP	0.329	**0.012**
AP	0.072	0.248
AK	0.385	**0.010**
EC	0.576	**0.001**

SOM, soil organic matter; TN, total nitrogen; TP, total phosphorus; AP, available phosphorus; AK, available potassium; EC, electrical conductivity. The bold number indicate a significant effect of soil variables on fungal communities.

### Fungal community composition and enriched OTUs

**Figure 3:**
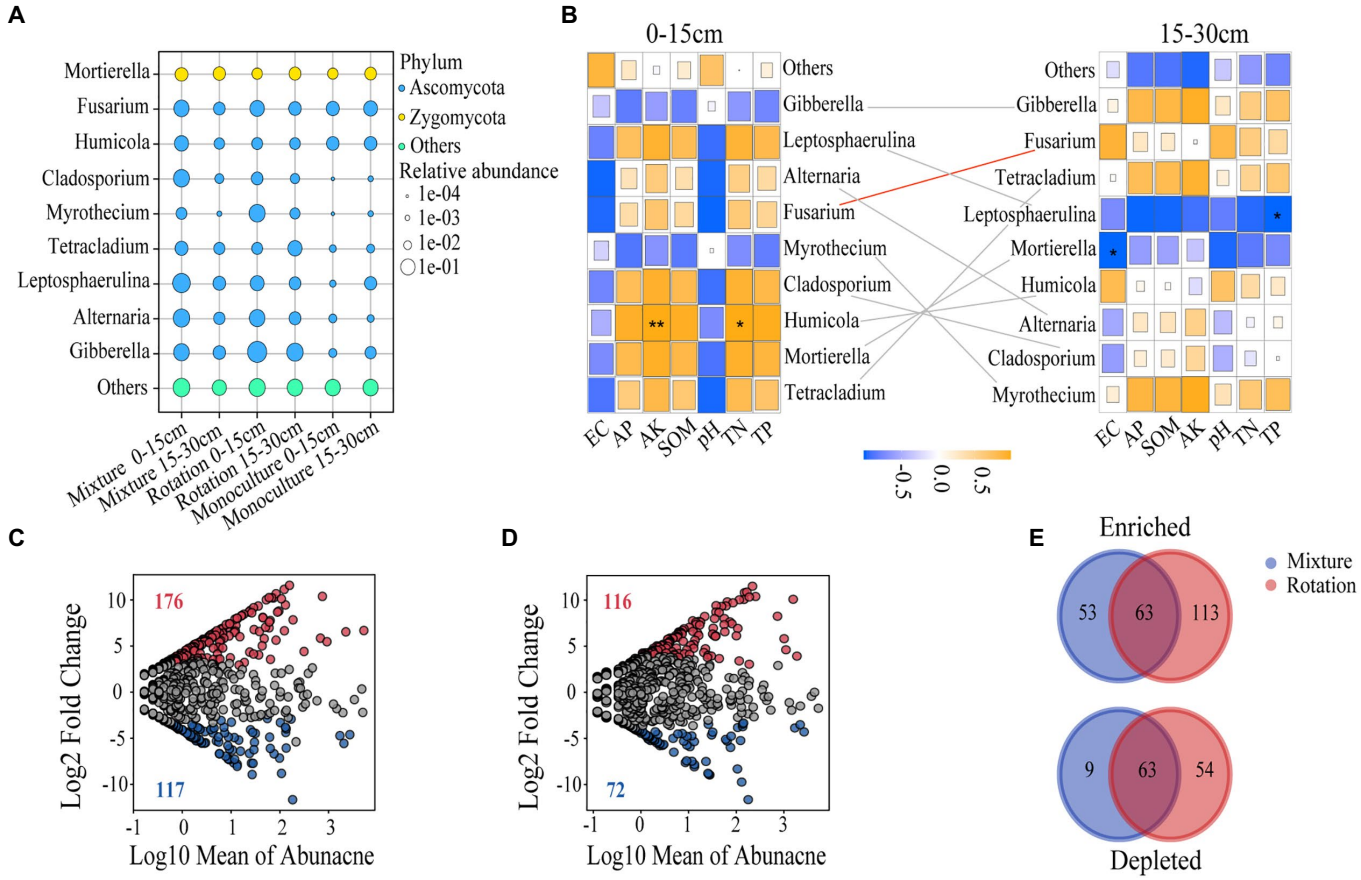
The bubble map shows the relative abundance of dominant genera in the fungal community **(A)**. Correlation heat maps show the correlation between dominant genera and soil chemical properties at 0–15 cm and 15–30 cm soil depths **(B)**. The red line indicates that soil depth influences the relative abundance of dominant genera, while gray has no effect. Enrichment and depletion of OTUs in rotation **(C)** and mixture **(D)** soils compared to monoculture. The number of enriched and depleted OTUs differed between each compartment **(E)**. *p ≤ 0.05; **p ≤ 0.01.

Because of the small number of known phyla in the soil fungal communities (Supplementary Figure 3), we analyzed the most abundant genera (Figure 3A). The relative abundance of *Leptosphaerulina* in the monoculture was significantly lower than that in mixture in the 0–15 cm soil layer (Figure 3A; Supplementary Table 3). The relative abundance of *Alternaria* in the mixture and rotation fields was significantly higher than that in the monoculture in the 15–30 cm soil layer (Figure 3A and Supplementary Table 3). Similarly, the relative abundance of *Myrothecium* in the monoculture and mixture was significantly lower than that in rotation at 15–30 cm soil layer (Figure 3A and Supplementary Table 3). Furthermore, the relative abundance of *Gibberella* in the rotation fields was greater than that of other cropping patterns in both soil layers (Figure 3A and Supplementary Table 3). The relative abundance of *Tetracladium* was highest in the mixture fields at 0–15 cm soil layer and in the rotation fields in the 15–30 cm soil layer, respectively (Figure 3A and Supplementary Table 3).

We also tested the effect of the soil chemistry on the dominant genera, and a Spearman correlation analysis was carried out (Figure 3B). In the 0–15 cm soil layer, the dominant genus, *Humicola*, was significantly positively correlated with AK and TN (Figure 3B). The dominant genera, *Leptosphaerulina* and *Mortierella*, were significantly negatively correlated with TP and EC in the 15–30 cm soil layer, respectively (Figure 3B). We also found that the relative of *Fusarium* was significantly affected by soil depth (Figure 3B and Supplementary Table 4).

A total of 116 OTUs showed significant enrichment in at least one compartment (Figures 3C,D and Supplementary Table 5). The number of enriched OTUs was greater than that of depleted OTUs (116 vs. 72), indicating an enrichment effect in the mixture fields (Figure 3D). Although many OTUs were enriched in the rotationfields, it simultaneously depleted a larger proportion of OTUs (176 vs. 117) (Figure 3C). Overall, both the rotation and mixture fields showed significant enrichment effects compared to the monoculture soils (Figures 3C,D). Mixture and rotation fields have enriched 63 overlapping OTUs, primarily consisting of the Ascomycota (Figure 3E and Supplementary Table 6). Similarly, 53 and 113 OTUs were uniquely enriched in the fungal communities of the mixture and rotation soils, respectively (Figure 3E). The mixture has 63 overlapping OTUs of the 117 depleted OTUs in the rotation, which mainly consisted of Ascomycota and Basidiomycota (Figure 3E and Supplementary Table 6). Similarly, the 9 and 54 OTUs were uniquely depleted in the fungal communities of mixture and rotation soils, respectively (Figure 3E).

### Soil fungal community assembly

**Figure 4:**
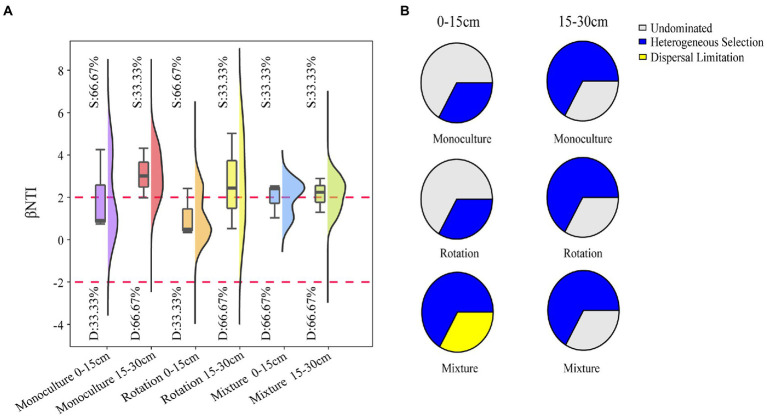
βNTI in different microhabitats across three cropping patterns at 0–15 cm and 15–30 cm soil depths **(A)**. Null model analysis of the community assembly ecological processes **(B)**.

We performed a null model to explore the fungal community assembly process across three cropping patterns (Figure 4 and Supplementary Figure 4). Monocultures and rotations were dominated by stochastic processes, whereas mixture systems were dominated by deterministic processes in the 0–15 cm soil layer (Figure 4A). However, the three cropping patterns were dominated by deterministic processes in the 15–30 cm soil layer (Figure 4A). For ecological processes, the stochasticity of the rotation and monoculture systems was driven by ecological drift, while the certainty of mixture systems was driven by heterogeneous selection in the 0–15 cm soil layer (Figure 4). The determinism of the three cropping patterns was driven by heterogeneous selection at 15–30 cm soil layer (Figure 4B). To further elucidate fungal community assembly, the neutral models were tested (Supplementary Figure 5). In total, 46, 45, and 38% of the variance was explained by the model in fungal communities for the monoculture, rotation, and mixture soils, respectively (Supplementary Figure 5). In addition, the estimated mobility (M) was 0.30, 0.28, and 0.09 in the monoculture, rotation, and mixture soils, respectively (Supplementary Figure 5).

### Fungal community co-occurrence network

**Figure 5:**
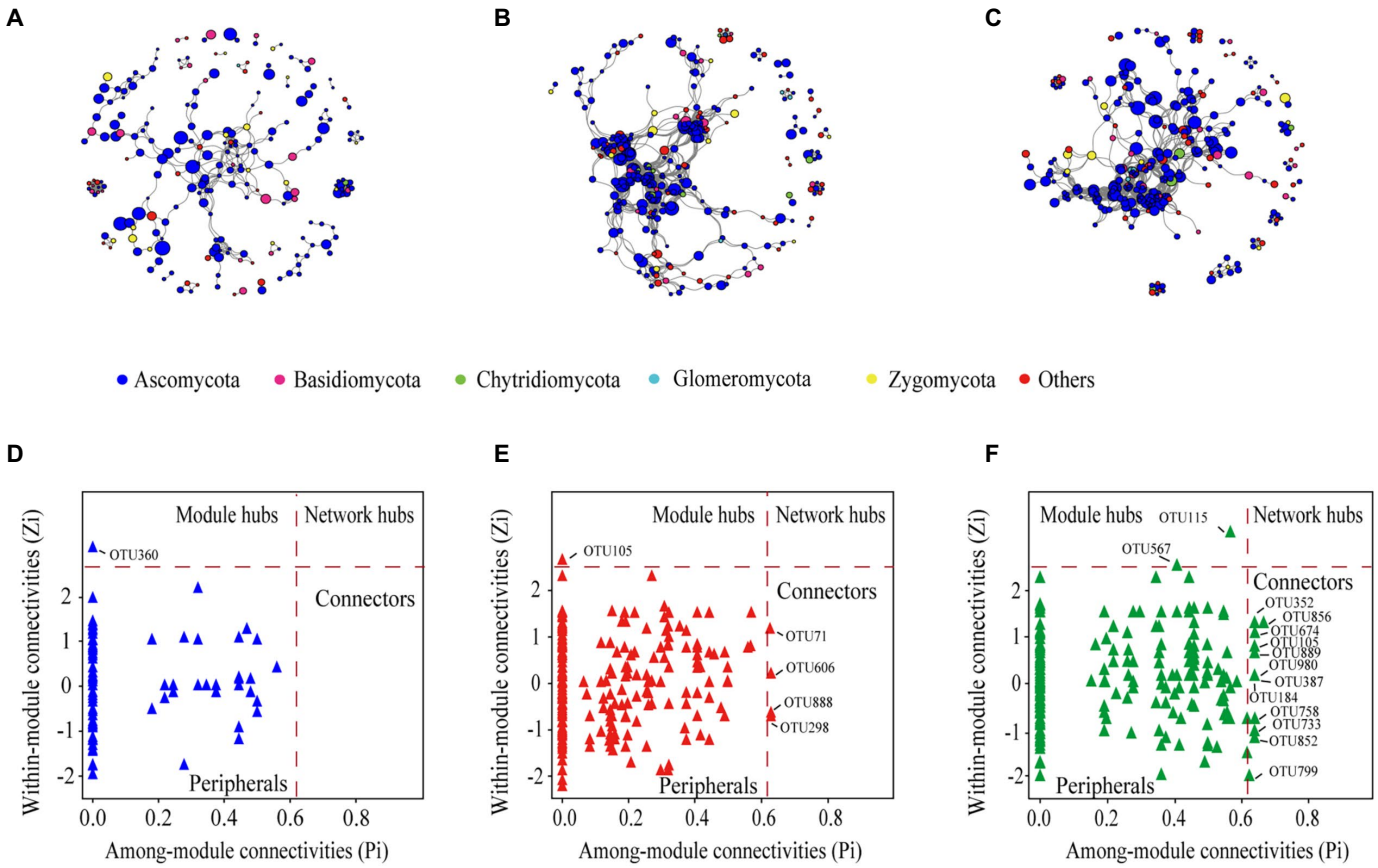
The co-occurrence network of soil fungal community in monoculture **(A)**, rotation **(B)**, and mixture **(C)**, the size of each node is proportional to its relative abundance. Zi-Pi plots showing the distribution of OTUs based on their topological roles in monoculture **(D)**, rotation **(E)**, and mixture **(F)**.

The fungal community networks and main topological properties between the three cropping patterns were analyzed (Figures 5A–C and Table 2). In terms of the number of nodes and lines, rotation and mixture have more complex networks than monoculture (Figures 5A–C and Table 2). These results also manifested in the network topological properties, namely, that the connectedness in rotation and mixture were two orders of magnitude larger than those in monoculture soils (Table 2). The phylum-level features also show a partition of the overall network in several clusters, with the monoculture having the greatest number of clusters (25), followed by the rotation (16) and mixture soils (18) (Table 2).

**Table 2:** Major topological properties of the co-occurrence network of soil fungal communities.

Topological properties	Monoculture	Rotation	Mixture
No. of original OTUs	273	324	337
Nodes	226	302	312
Edges	523	1887	1970
Average degree	0.02	12.50	12.63
No. of clusters	25	16	18
clustering coefficient	0.78	0.62	0.57
Average path distance	7.45	4.93	4.46
Connectedness	0.02	0.04	0.04
Positive links	518	1815	1945
Negative links	5	72	25

More module hubs and connectors were noted in the Zi and Pi plot in the crop rotation and mixture networks than in the monoculture (Figures 5D–F). One key note was found in monocultures, belonging to Ascomycota; five key notes were found in rotation networks, belonging to Ascomycota; and 14 key notes were found in mixture networks, belonging to Chytridiomycota, Zygomycota, and Ascomycota. The phylogenetic classification of the hubs and connectors of each module is shown in Supplementary Table 7.

### Fungal community functional groups

**Figure 6:**
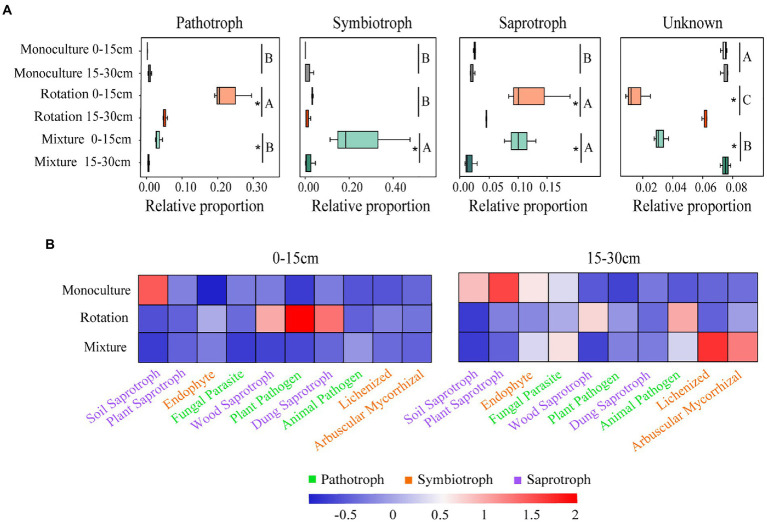
Variations in fungal function **(A)** and composition of fungal functional groups (guilds) inferred by FUNGuild **(B)**. Different letters indicate significant differences between groups (*p* < 0.05). A significant difference between soil depths and statistical significance is indicated as follows. *p ≤ 0.05.

In general, 6.67, 79.36, and 55.52% of OTUs in the monoculture, rotation, and mixture soils, respectively, were established as nutrient patterns with saprotroph, pathotroph, and symbiotroph while the remainder were unallocated in the 0–15 cm soil layer (Figure 6A and Supplementary Table 8). In the 15–30 cm soil layer, 6.98, 21.57, and 6.38% of OTUs in monoculture, rotation, and mixture soils, respectively, were established as nutrient patterns with saprotroph, pathotroph, and symbiotroph, while the remainder were unallocated (Figure 6A and Supplementary Table 8). The relative proportion of pathotroph, symbiotroph, and saprotroph was affected by both cropping patterns and soil depth (Supplementary Table 9). Of these, the relative abundances of pathotroph in the rotation system and the relative of symbiotroph in mixture system were highest among the three cropping patterns (Figure 6A). The relative proportion of saprotroph in the rotation and mixture system was higher than that in monoculture system (Figure 6A). The relative proportion of saprotroph and pathotroph in the rotation system was significantly affected by soil depth (Figure 6A). The relative proportion of three nutrient patterns in the mixture system was significantly affected by soil depth (Figure 6A). In addition, we found a significant negative correlation between the Shannon index and the relative abundance of pathotroph in rotation soils (Supplementary Figure 6). In the same way, more detailed information of nutritional patterns was obtained (Figure 6B and Supplementary Table 10). The lichenized relative abundance was significantly higher in the mixture system in the 0–15 cm soil layer compared to the other crop systems (Figure 6B and Supplementary Table 10). The relative abundance of soil saprotroph was significantly higher in the monoculture in the 15–30 cm soil layer compared to the other crop systems (Figure 6B and Supplementary Table 10). The relative abundance of plant pathogen and plant saprotroph was significantly higher in rotation and monoculture soils, respectively, compared to the other cropping systems (Figure 6B and Supplementary Table 10). These results indicate that cropping types and soil depth have an effect on the fungal community function.

## Discussion

### Cropping patterns affected fungal community structure

Our study shows that rotation and mixture systems promote the recovery of saline-alkali soils in the Western Songnen Plain, northeastern China. Compared to the monoculture system, soil pH in the mixture system was decreased by 5.74 and 6.42% in the 0–15 cm and 15–30 cm soil layers, respectively. Soil pH in the rotation was decreased by 2.56 and 1.33% at 0–15 cm and 15–30 cm depths, respectively (Supplementary Table 1). Mixture and rotation increase root secretions containing organic acids and neutralize CO_3_^2−^ and HCO_3_^−^ content on soil colloids (Guo et al., 2018). Plant capacity to reduce soil salinity relies on numerous factors, such as direct absorption of salt ions, storage in cells and tissues, or secretion of excess salt through salt glands or salt bladders (Hasanuzzaman et al., 2014; Yuan et al., 2016). Oats and tall wheat grass were grown into mixed crop and rotation systems. Oat stalks can absorb soil salts and harvest the straw of oat, which can reduce soil salt content (Wang and Song, 2006). Tall wheatgrass has been reported to be salt tolerant and to have the ability to reduce soil pH (Ludwig et al., 1978). In addition, it has also been demonstrated that the Conovolvulaceae (alfalfa) and Poaceae (oats and tall wheatgrass) families have salt gland structures that secrete salt from inside of the plant into the surface of the plant’s stems and leaves (Yuan et al., 2016). Rotation can also improve soil nutrients through the continuous accumulation of crop residues, and a favorable soil nutrient status can inhibit the accumulation of soil salts (Zhang, 1998). In addition, salt transport and distribution in the soil can be influenced by the evaporation of soil water (Shokri-Kuehni et al., 2017). Mixed cropping systems can reduce the evaporation of soil water and likewise inhibit the accumulation of salts. The findings from the present study indicated that soil pH exhibited a negative effect on OTU richness (Figure 2B). Compared to the monoculture, rotation and mixture system had a better ability to reduce salt and soil pH and promoted the OTU richness of fungal communities.

Plants influence the diversity of soil fungal communities by providing large amounts of carbon and energy resources through photosynthesis for the growth of soil fungi (Requena et al., 1997; Bruggen and Semenov, 2000; Sláviková et al., 2002; Jean-Franois, 2013). Mixed cropping systems increase carbon input to soils due to the dominance of above-ground plant biomass, which could promote an increase in the OTU richness of fungal communities. In addition, the residue from previous crop was left on the soil surface as green manure for long-term soil restoration (Peltoniemi et al., 2021), which may have contributed to the increase in soil carbon and OTU richness of the fungal communities. In this study, mixture and rotation systems promoted the richness of the fungal community by increasing carbon input to the soil, which in turn increased the efficiency of nutrient decomposition by soil fungi and promoted the restoration of nutrients in saline-alkali soils. The diversity of fungal communities has an critical impact on crop and soil chemistry (Rousk et al., 2010; Duchicela et al., 2013; Bender et al., 2014; van der Heijden et al., 2016). Saline-alkali soil properties showed a similar trend compared to changes in fungal diversity, suggesting a relationship between fungal diversity and soil properties (Supplementary Table 1). Higher fungal diversity increases the rate of soil nutrient decomposition, resulting in changes in the fungal diversity that were similar to the overall trend of soil nutrients (e.g., TN, TP, and AK) (Kivlin et al., 2014; Hiscox et al., 2015; Yao et al., 2017b). We have confirmed this by building a linear regression model (Supplementary Figure 2). A previous study has shown that AK significantly affects the structure of fungal communities, such as abundance, diversity, and composition (Luo, X. et al., 2021). Soil N is a key factor for crop growth, and several studies have indicated that TN plays a key role in the diversity of fungal community (Wu et al., 2015; Zeng et al., 2019; Chen et al., 2021). TN significantly positively contributes to the richness and diversity of fungal communities (Liu J. J. et al., 2021). We found that mixture and rotation system may have influenced the changes in the Shannon index and OTU richness of fungal communities by promoting the increase in AK and TN content, respectively. The AK and TN content in mixture and rotation system were different forms in the two soil depths, which confirms the variation of the Shannon index and OTU richness of fungal communities (Figure 2). The changes in TN content of mixture system in the 0–30 cm soil layer (Supplementary Table 1), which may be why soil depth affects OTU richness (Figure 2B).

In addition, EC is a key factor influencing changes in fungal communities of improved and poor soils (Wang et al., 2020). Zeng et al. (2020) reported that excessive EC repressed the microbial activity and severely affected the gradient succession of fungal communities in the arid loess region of China. Mixture and rotation systems alleviated the salt stress on the fungal community by reducing the soil EC, which in turn promoted the increase of OTU richness in the fungal community (Figure 2B and Supplementary Table 1). Subsequently, we further found that TP also had a significant effect on fungal community structure using a Mantle test (Table 1). As with soil nitrogen, the availability of soil phosphorus can impact the occurrence of microbe–plant symbioses, which in turn can influence the microbial community in roots and soils (Ren et al., 2021). As the sampling site area is in saline-alkali soils, phosphorus occurs in the form of Ca-P and is not absorbed by plants (Li et al., 2015). Plants will recruit and select microbial groups with specific phosphorus-solubilizing functions to alleviate phosphorus deficiency (Lu and Huang, 2010). In addition, roots secrete organic acids associated with dissolved phosphorus to enhance phosphorus utilization, thus influencing microbial communities (Masaoka et al., 1993; Jones et al., 2004). Fungal richness decreases as a result of a reduction in SOM with increasing soil depths (Gittel et al., 2014), which may explain the differences in the beta diversity index for the mixed crop system in the two soil layers (Supplementary Table 1 and Figure 2D). Soil nutrients also create a suitable growth environment for fungal activity, and the two complement each other (Landeras et al., 2005; Baldrian et al., 2012; Sun et al., 2015). Overall, our results confirm our first hypothesis that complex planting restoration patterns will lead to increased diversity of soil fungal communities.

Our second hypothesis is that complex cropping patterns used for saline-alkali restoration can cause changes in fungal community composition, which is supported by the results of this study. Compared with the monoculture, the rotation system had higher relative abundances of *Gibberella* in two soil layers. The availability of oxygen is essential for the growth of *Gibberella* (Weizhong, 2003). A previous study reported that rotation significantly increased soil porosity by 3.2 to 6.7% and that the greater availability of oxygen contributed to an increase in the relative abundance of *Gibberella* (Zhang et al., 2021). The relative abundance of *Tetracladium* was the highest in the mixture system in the 0–15 cm soil layer and in rotation at the 15–30 cm soil layer, respectively (Figure 3A and Supplementary Table 3). It has been reported that *Tetracladium* has a considerable ability to decay organic substrates and that changes in fungal communities strongly correlate with variations in carbon resources in the soil (Abdel-Raheem, 1997; Anderson and Marvanová, 2020; Sun et al., 2020). The variation in SOM in our study was consistent with changes in the relative abundance of *Tetracladium* (Supplementary Table 1). In addition, the rotation system significantly increased the relative abundance of *Myrothecium* relative abundance, which has been confirmed by recent studies (Jin et al., 2019; Jia et al., 2020). In rotation and mixed crop systems, *Alternaria* was increased significantly (Figure 3A and Supplementary Table 3). Because they regulate the energy input, plants have a direct impact on the soil microbial community (Wiesner et al., 2020). The composition of the above-ground organs may influence the composition of the soil fungal community through plant residues and rhizodeposition (Gong et al., 2016; Persoh et al., 2018). The *Leptosphaerulina* was negatively correlated with aflatoxin (AFTs)-producing fungi abundance and may inhibit the growth of AFTs-producing fungi (Qi et al., 2022). We therefore speculated that mixture system may reduce the risk of AFTs and improve plant quality by increasing the relative abundance of *Leptosphaerulina*. Furthermore, we also found that the genera with significant changes in fungal community composition all seemed to come from the Ascomycota, which is consistent with the phylum of overlap OTU with significant enrichment in rotation and mixture soils (Figure 3E and Supplementary Table 6). The Ascomycota had an essential role in the decomposition of the apoplast, which provides a direct source of energy for itself (Zheng et al., 2021). The Basidiomycota was also present in overlapping OTU that were significant depleted (Figure 3E and Supplementary Table 6). A previous study has indicated that Basidiomycota responds sensitively and changes distinctly in abundance with field management practices (Yu et al., 2021). It has been shown that integrated agricultural practices reduce the relative abundance of Basidiomycota (Zhu et al., 2018). The significant correlation between the different dominant genera and soil properties suggests that environmental conditions may strongly influence the composition of fungal communities (Yang et al., 2020; Han et al., 2021).

### Mixed crop system improve selection for fungal community assembly

In consistent to our hypothesis, a change in the community assembly was only found in soils from the mixed crop system (Figure 4A). The alternation of community composition relies on selection, dispersal, and drift in a metacommunity (Stegen et al., 2012). Our study shows that drift processes was present in fungal communities in rotation and monoculture systems, whereas the mixture system showed, a heterogeneous selection in the 0–15 cm soil layer (Figure 4B). Drift is the core of fungal community assembly (Sheng et al., 2019). As plant diversity increases and input soil nutrients, the dominance of deterministic processes leads to a community well adapted to environmental conditions (Ernakovich et al., 2022). These findings are supported by the results of the neutral model, which indicated that the mixture system led to higher environmental selection, indicating that a reduced degree of dispersal resulted from biological factors associated with increasing plant richness (Supplementary Figure 5). Conversely, stochastic processes increase the number of maladapted taxa and may reduce ecosystem function for some microbiomes (Ernakovich et al., 2022). In addition, selection would lead to more similar phylogenetic groups and make their communities better aligned against external disturbances (Yu et al., 2021). Soil nutrient enrichment resulting from mixture system reduces the importance of community stochasticity and imposes a deterministic environmental filter, to a degree allowing that fungal communities ecological niche-based selection (Conradi et al., 2017).

### Fungal networks of rotation and mixed crop systems show greater complexity

Because most microorganisms are unable to survive independently, microorganisms often build complex ecological networks to coexist (Faust et al., 2015) and often rely on each other’s extracellular metabolites for survival (Pande and Kost, 2017). In this study, we discovered that complex cropping types (i.e., rotation and mixture system) of species built more connections than monocultures (Table 2), suggesting that the OTUs of rotation and mixture systems can more easily establish a mutually beneficial community. A highly connected network has been suggested to provide more functional redundancy (Mougi and Kondoh, 2012). This indicates that the networks in the rotation and mixture systems networks would lead to stronger resistance to disturbance and improved community stability (Scheffer et al., 2012). In addition, a more connected network can more efficiently utilize carbon and improve nutrient exchange between various species (Morrien et al., 2017). Soil carbon availability is significantly correlated with fungal community composition and greater resource availability is thought to reduce competition in microbial communities (Hubbell, 2005; Costello et al., 2012; Loganathachetti et al., 2017). This implies that mixture and rotation systems are more suitable than monocultures for the restoration of ecological networks of fungal communities in saline-alkali soils.

Saprotrophic fungi favor C and nutrient-rich niches and are more efficient in utilizing energy-rich litter (Crowther et al., 2012; Chen et al., 2019); therefore, they are recommended as r-strategists (Li et al., 2021). Most saprotrophic fungi fall within the phyla of Ascomycota and Zygomycota have been categorized as r-members (Yao et al., 2017a). Similarly, Chytridiomycota also contains saprotrophic fungi and is commonly considered an r-member (Van den Wyngaert et al., 2022). The Ascomycota, the largest fungal phylum, which has been found to be widely distributed in soil, may be the most important phylum in saline restoration ecosystems (Fierer, 2017). Fierer and Jackson (2006) reported that the community of microorganisms was divided into r-strategic symbiotic microbes and k-strategic oligotrophic microbes. Compared to monocultures, composite planting types have higher plant richness, and soil fungi are active in environments with high nutrient availability and low-stress exposure, but have low survival rates (r-selected) (Pianka, 1970; Ramirez et al., 2012). Thus, rotation and mixture systems promoted the abundance of the key fungal taxa Ascomycota and Zygomycota. In addition, the greater relative abundance of Ascomycota also contributes to fungal richness (Nevins et al., 2021). Interestingly, we found that the Chytridiomycota and Zygomycota were keystone taxa in mixed crop systems, which were not found in the other two cropping patterns (Figure 5F and Supplementary Table 7). The Zygomycota was significantly associated with physical C protection, implying that networks in the mixed crop system are more conducive to soil carbon sequestration (Zhu et al., 2021). In addition, the Chytridiomycota can promote nutrient cycling in terrestrial ecosystems (Hanrahan-Tan et al., 2022). Soil networks in the mixed crop system are superior to those in monocultures and rotation systems, both in terms of network stability and ecosystem health.

### Cropping patterns change the nutritional patterns of fungal communities

Previous studies have shown that rotation systems increase the relative abundance of pathogenic fungi, leading to a significant decrease in productivity (Du et al., 2022; Jin et al., 2022). With the increase in the number of crop rotations and planting years, the soil quality of long-term rotational plots will be reduced (Götze et al., 2017). This will lead to ecological imbalance of rhizosphere soil microorganisms, a change in the population structure, and a reduction of beneficial microorganisms (Du et al., 2022). Subsequently, pathogenic microorganisms are enriched and are more likely to grow in the rhizosphere soil, which cause various soil-borne diseases (Seker et al., 2017). A soil microbial community with high diversity is less susceptible to pathogen invasion and can reduce the probability of soil-borne diseases (Wei et al., 2018, 2019). Previous studies have suggested that the reduced diversity of microbial communities in soils that have been under long-term rotation cultivation is due to pathogenic fungi occupying a dominant position in rhizosphere soil communities as well as interspecific competition, while some of the corresponding beneficial fungi may be eliminated due to weak competitiveness (Siegel-Hertz et al., 2018). In our study, the relative abundance of pathogenic fungi in the rotation system was significantly negatively correlated with the Shannon index (Supplementary Figure 6). In addition, cover crops provide a greater source of organic carbon and will create a richer community of symbiotic fungi (Schmidt et al., 2019). The highest relative abundance of symbiotic fungal communities in mixture system may be due to the enrichment of soil organic carbon. The distribution of lichenized fungi categorized as symbiotic fungi was also correlated with plant cover (Liu Y.-R. et al., 2021). Furthermore, Gao et al. (2022) highlighted that soil moisture suppressed the abundance of symbiotic fungi. Compared to the other two cropping types, mixtures soils use less shallow soil water (O’Keefe et al., 2019). It is well established that symbiotic fungi may have important benefits for the health, nutrition, and overall quality of crops (Sagan and Margulis, 1999; Rouphael et al., 2015; Igiehon and Babalola, 2017). The quality and availability of substrate in the soil is a key factor in determining the role of saprophytic fungi in the decay of soil litter and SOM (Maranon-Jimenez et al., 2021). In the 0–15 cm soil layer, mixture soils may be healthier and have better ecosystem function than rotation and monoculture soils. In addition, the biomass of saprophytic fungi appears to drive a strong positive correlation between fungal biomass and soil carbon (Whalen et al., 2021). Saprophytic fungi, a key microbial group, mediate nutrient cycling in soil ecosystems by releasing lignocellulolytic enzymes to break down complex organic matter (Crowther et al., 2012). Rotation and mixture systems promote the activity of saprophytic fungi by increasing the input of soil carbon, while saprophytic fungi also promote the decomposition of organic matter. Therefore, the nutrient cycling of rotation and mixture systems was better than that of monoculture soils. Unexpectedly, we observed higher abundance of plant saprophytes and soil saprophytes in monoculture than that of mixture and rotation soils, which may be due to the presence of more unknown saprophytic fungi in mixture and rotation soils. As FUNGuild is based on a comparison of existing databases, the function of complex fungal communities still needs to be studied in depth (Wang et al., 2018). Overall, the ecological functionality of rotation and mixture soils is better than that of monocultures.

## Conclusion

This study showed that mixture and rotation systems could promote the recovery of nutrients in saline-alkali soils of the Western Songnen Plain, northeastern China, due to the growth of oats and tall wheatgrass. Rotation and mixtures systems had a better desalination effect than the monoculture system, and the desalination effect was better in the 0–15 cm soil layer than in the 15–30 cm soil layer. The mixed crop and rotation systems promoted the richness of fungal communities by increasing the carbon input into the soil. The composition, structure, and functionality of soil fungal communities differed significantly between the three cropping patterns. In addition, rotation and mixture systems enhance positive interactions between fungi and improve community stability. The fungal network in the mixed crop system is more connected than the fungal network of monoculture and rotation systems in terms of key taxa and ecosystem health. On a local scale, the increased plant species richness provides more ecological niches, allowing the mixture system in the 0–15 cm soil layer to be dominated by deterministic processes. It is important to note that the relative abundance of pathogenic fungi, such as *Gibberella*, increases under the rotational cropping pattern, and therefore the threat of soil disease should be a concern when considering saline restoration methods. This study found that rotation and mixture systems promoted the restoration of fungal communities in saline-alkali soils, and that mixture system was preferred over the rotation system.

## Data availability statement

The datasets presented in this study can be found in online repositories. The names of the repository/repositories and accession number(s) can be found in the article.

## Author contributions

BL: methodology, data curation, conceptualization, writing—original draft, and writing—review and editing. XQL: methodology and formal analysis. DZ: data curation and formal analysis. HS: methodology and data curation. KG: conceptualization, methodology, and data curation. GS: project administration. XNL: conceptualization, funding acquisition, and project administration. LS: conceptualization and methodology. All authors contributed to the article and approved the submitted version.

## Funding

This study was funded by the Opening Project of State Key Laboratory of Tree Genetics and Breeding (K2021103), Academic backbone Support Project of the Northeast Agricultural University, Postdoctoral Science Foundation (2018 M640287), and the National Natural Science Foundation of China (41701289).

## Conflict of interest

The authors declare that the research was conducted in the absence of any commercial or financial relationships that could be construed as a potential conflict of interest.

## Publisher’s note

All claims expressed in this article are solely those of the authors and do not necessarily represent those of their affiliated organizations, or those of the publisher, the editors and the reviewers. Any product that may be evaluated in this article, or claim that may be made by its manufacturer, is not guaranteed or endorsed by the publisher.
