# Supplementary material for: Crop diversity promotes the recovery of fungal communities in saline-alkali areas of the Western Songnen Plain
Source: Front Microbiol. 2023 Feb 1;14:1091117. doi: 10.3389/fmicb.2023.1091117 (PMC9930164; doi:10.3389/fmicb.2023.1091117)
Supplement: Supplementary file 1 [file Table_1.docx]

**Supplementary Figure 1** Distribution of fungal communities OTUs at 0-15 cm (A) and 15-30 cm (B) soil depths.

**Supplementary Figure 2** Linear regressions between Shannon diversity index (A) and OTU richness and soil chemical properties (B).

**Supplementary Figure 3** The relative abundance of fungal community composition for three cropping patterns at the phylum level at 0-15 cm (A) and 15-30 cm (B) soil depths.

**Supplementary Figure 4** Raup-Crick index distribution of fungal communities of three cropping patterns at 0-15 cm (A) and 15-30 cm (B) soil depths.

**Supplementary Figure 5** Fit of Sloan's neutral model for the analysis of monoculture (A), rotation (B), and mixture (C) community assembly processes. The solid black lines indicate the best fit to the model and the dashed lines represent the 95% confidence intervals around the best‐fitting neutral model. Blue solid circle presents OTUs that fit the model. Red and yellow solid circles represent OTUs that had higher and lower abundances than predicted, respectively. M indicates the metacommunity size multiplied by immigration, while R^2^ indicates model fit.

**Supplementary Figure 6** Linear regressions between Shannon diversity index and the relative of pathotroph in rotation soils.
