# Supplementary material for: Crop diversity promotes the recovery of fungal communities in saline-alkali areas of the Western Songnen Plain
Source: Front Microbiol. 2023 Feb 1;14:1091117. doi: 10.3389/fmicb.2023.1091117 (PMC9930164; doi:10.3389/fmicb.2023.1091117)
Supplement: Supplementary file 2 [file Table_2.docx]

**Supplementary Table 1**. The chemical properties of soils in the three groups.

**Supplementary Table 2**. Two-way ANOVAs for the impact of cropping patterns (CP), soil depth (SD) and their interaction (PT × SD) on the Shannon index, OTU richness, and beta diversity index.

**Supplementary Table 3**. The effects of cropping patterns on the relative abundance of dominant genera in fungal communities at two soil depths.

**Supplementary Table 4**. Effect of soil depth on the relative abundance of dominant genera in fungal communities by T-test.

**Supplementary Table 5**. Differentially abundant OTUs are test by fitted negative binomial generalised log-linear models among three cropping patterns.

**Supplementary Table 6**. Each significantly enriched and declined OTUs at the taxonomic level.

**Supplementary Table 7**. The classification at each taxonomic level of the generalists.

**Supplementary Table 8**. Relative proportion (%) of fungal function from selected soil samples inferred by FunGuild.

**Supplementary Table 9**. Two-way ANOVAs for the impact of cropping patterns (CP), soil depth (SD) and their interaction (CP × SD) on the functional trophic mode inferred by FunGuild.

**Supplementary Table 10**. Compositions and relative abundance (%) of fungal functional group (guild) from selected soil samples inferred by FunGuild.

**Supplementary Table 1**.

|  | Monoculture | |  | Rotation | |  | Mixture | |
| --- | --- | --- | --- | --- | --- | --- | --- | --- |
|  | 0-15cm | 15-30 cm |  | 0-15 cm | 15-30 cm |  | 0-15 cm | 15-30 cm |
| pH | 8.19±0.08 | 8.26±0.13 |  | 7.98±0.14 | 8.15±0.06 |  | 7.72±0.11 | 7.73±0.20 |
| SOM | 49.02±1.87 | 48.78±2.44 |  | 42.05±8.85 | 50.95±14.84 |  | 55.44±1.06 | 46.81±2.14 |
| TP | 0.54±0.21 | 0.39±0.00 |  | 0.37±0.09 | 0.43±0.05 |  | 0.73±0.35 | 0.31±0.33 |
| TN | 2.74±0.99 | 2.11±0.40 |  | 2.41±0.24 | 2.15±0.17 |  | 3.42±0.20 | 1.91±0.15 |
| AP | 61.95±11.53 | 49.16±2.07 |  | 52.31±14.57 | 58.57±0.93 |  | 69.24±29.00 | 39.31±4.35 |
| AK | 50.01±4.23 | 43.61±0.04 |  | 41.48±6.05 | 51.61±3.20 |  | 71.85±8.80 | 40.95±0.92 |
| EC | 417.84±1.95 | 443.23±12.80 |  | 392.63±3.03 | 440.75±20.31 |  | 382.32±2.49 | 435.98±6.47 |

**Supplementary Table 2**.

|  |  | Shannon diversity | |  | OTU richness | |  | Beta diversity | |
| --- | --- | --- | --- | --- | --- | --- | --- | --- | --- |
|  |  | *F* | *P* |  | *F* | *P* |  | *F* | *P* |
| CP |  | 3.401 | 0.056 |  | 6.103 | **0.015** |  | 2.156 | 0.159 |
| SD |  | 0.136 | 0.873 |  | 5.640 | **0.035** |  | 13.601 | **0.003** |
| CP 🞩SD |  | 3.884 | **0.019** |  | 13.343 | **<0.001** |  | 0.804 | 0.470 |

**Supplementary Table 3**.

| Genus | 0-15 cm | |  | 15-30 cm | |  |
| --- | --- | --- | --- | --- | --- | --- |
|  | *F* | *P* |  | *F* | *P* |  |
| *Gibberella* | 41.334 | **<0.001** |  | 125.902 | **<0.001** |  |
| *Leptosphaerulina* | 4.594 | **0.027** |  | 0.101 | 0.905 |  |
| *Alternaria* | 1.555 | 0.286 |  | 9.234 | **0.015** |  |
| *Fusarium* | 2.455 | 0.166 |  | 4.520 | 0.063 |  |
| *Myrothecium* | 1.178 | 0.370 |  | 20.342 | **0.002** |  |
| *Cladosporium* | 1.118 | 0.387 |  | 2.907 | 0.131 |  |
| *Humicola* | 2.147 | 0.198 |  | 2.382 | 0.173 |  |
| *Tetracladium* | 27.597 | **0.001** |  | 5.338 | **0.047** |  |
| *Mortierella* | 2.841 | 0.136 |  | 0.785 | 0.498 |  |
| Others | 36.470 | **<0.001** |  | 40.783 | **<0.001** |  |

**Supplementary Table 4**.

| Genus | Soil depth | |  |
| --- | --- | --- | --- |
|  | *F* | *P* |  |
| *Gibberella* | 20.829 | 0.107 |  |
| *Leptosphaerulina* | 6.639 | 0.149 |  |
| *Alternaria* | 10.206 | 0.082 |  |
| *Fusarium* | 3.966 | **0.041** |  |
| *Myrothecium* | 4.930 | 0.301 |  |
| *Cladosporium* | 4.977 | 0.287 |  |
| *Humicola* | 3.821 | 0.185 |  |
| *Tetracladium* | 4.467 | 0.430 |  |
| *Mortierella* | 2.155 | 0.642 |  |

**Supplementary Table 8**.

|  | Symbiotroph |  | Saprotroph |  | Pathotroph |  | Other |
| --- | --- | --- | --- | --- | --- | --- | --- |
| Monoculture 0-15cm | 16±8.72b |  | 2900.00±218.86b |  | 42.67±36.30c |  | 41403.00±1267.94a |
| Monoculture 15-30cm | 367.67±596.12b |  | 2389.33±505.55b |  | 376.00±381.91c |  | 41723.00±1538.81a |
| Rotation 0-15cm | 893.67±83.07b |  | 14625.67±6692.50a |  | 17345.33±4236.53a |  | 8545.67±4824.58d |
| Rotation 15-30cm | 296.33±333.20b |  | 5274.00±91.10b |  | 3861.67±512.91b |  | 34304.33±1065.78b |
| Mixture 0-15cm | 7051.00±5271.74a |  | 12014.00±3198.54a |  | 2992.67±796.22bc |  | 17670.33±2747.86c |
| Mixture 15-30cm | 482.33±683.36b |  | 1924.00±1321.72b |  | 445.66±184.19c |  | 41816.67±1967.50a |

**Supplementary Table 9**.

|  |  | Pathotroph | |  | Saprotroph | |  | Symbiotroph | |
| --- | --- | --- | --- | --- | --- | --- | --- | --- | --- |
|  |  | *F* | *P* |  | *F* | *P* |  | *F* | *P* |
| CP |  | 43.64 | **<0.001** |  | 9.799 | **0.003** |  | 6.376 | **0.013** |
| SD |  | 30.669 | **<0.001** |  | 25.687 | **<0.001** |  | 6.607 | **0.025** |
| CP🞩SD |  | 19.303 | **<0.001** |  | 5.584 | **0.019** |  | 5.897 | **0.016** |
