# Supplementary figures and images for: Crop diversity promotes the recovery of fungal communities in saline-alkali areas of the Western Songnen Plain
Source: Front Microbiol. 2023 Feb 1;14:1091117. doi: 10.3389/fmicb.2023.1091117 (PMC9930164; doi:10.3389/fmicb.2023.1091117)

A

Mixture

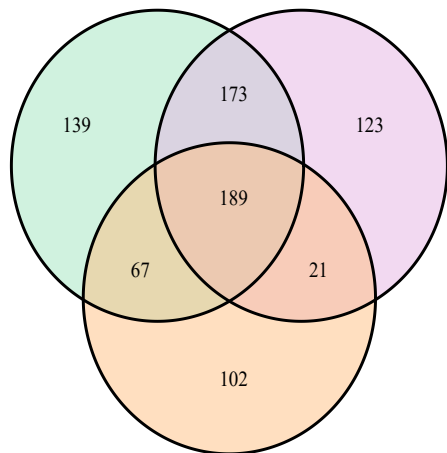

Monoculture

B

Mixture

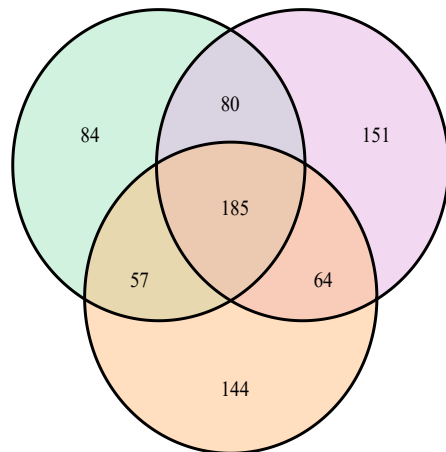

Rotation

Monoculture

Supplement: Supplementary file 4 [file Image_1.pdf]

A

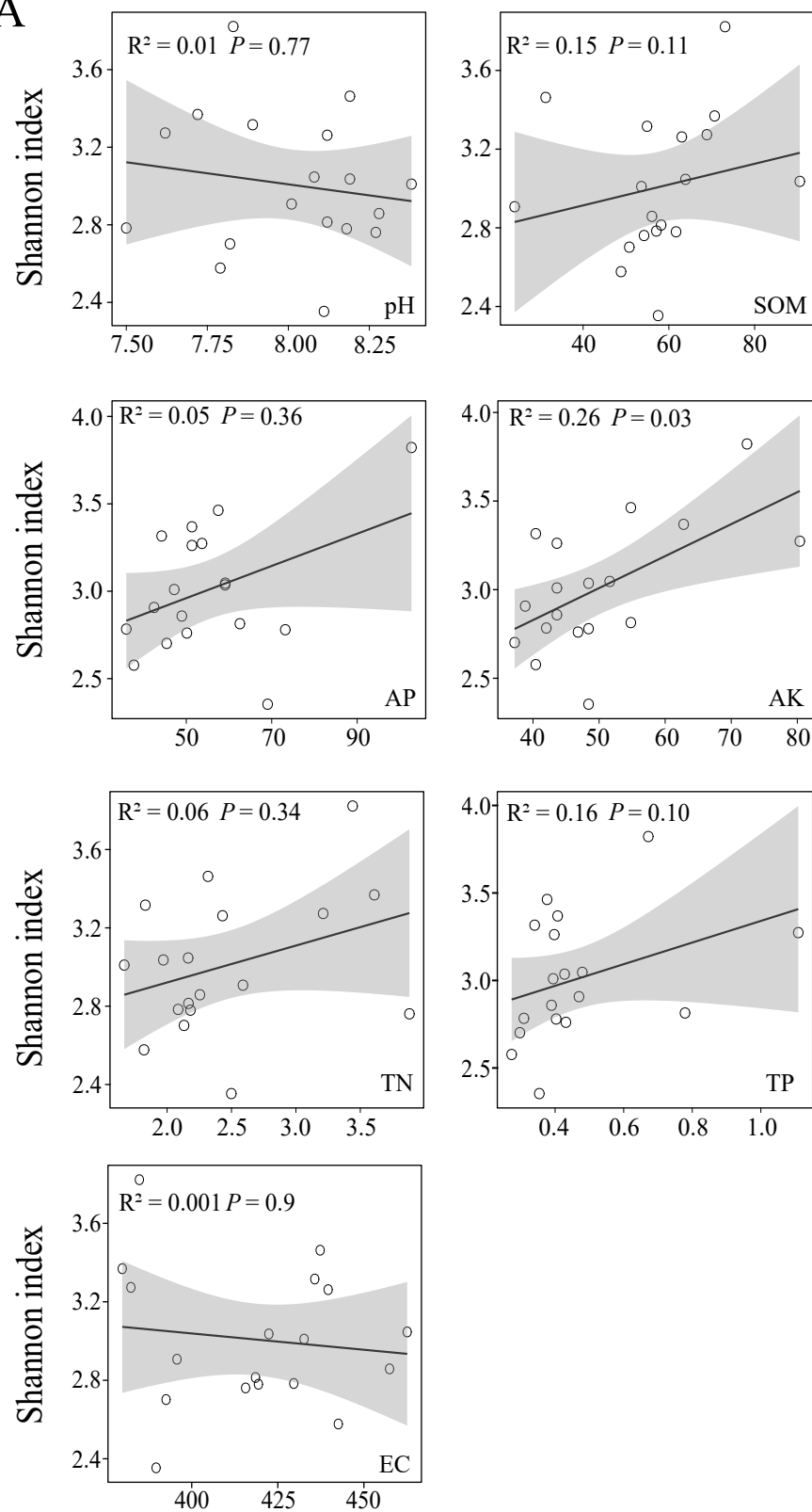

B

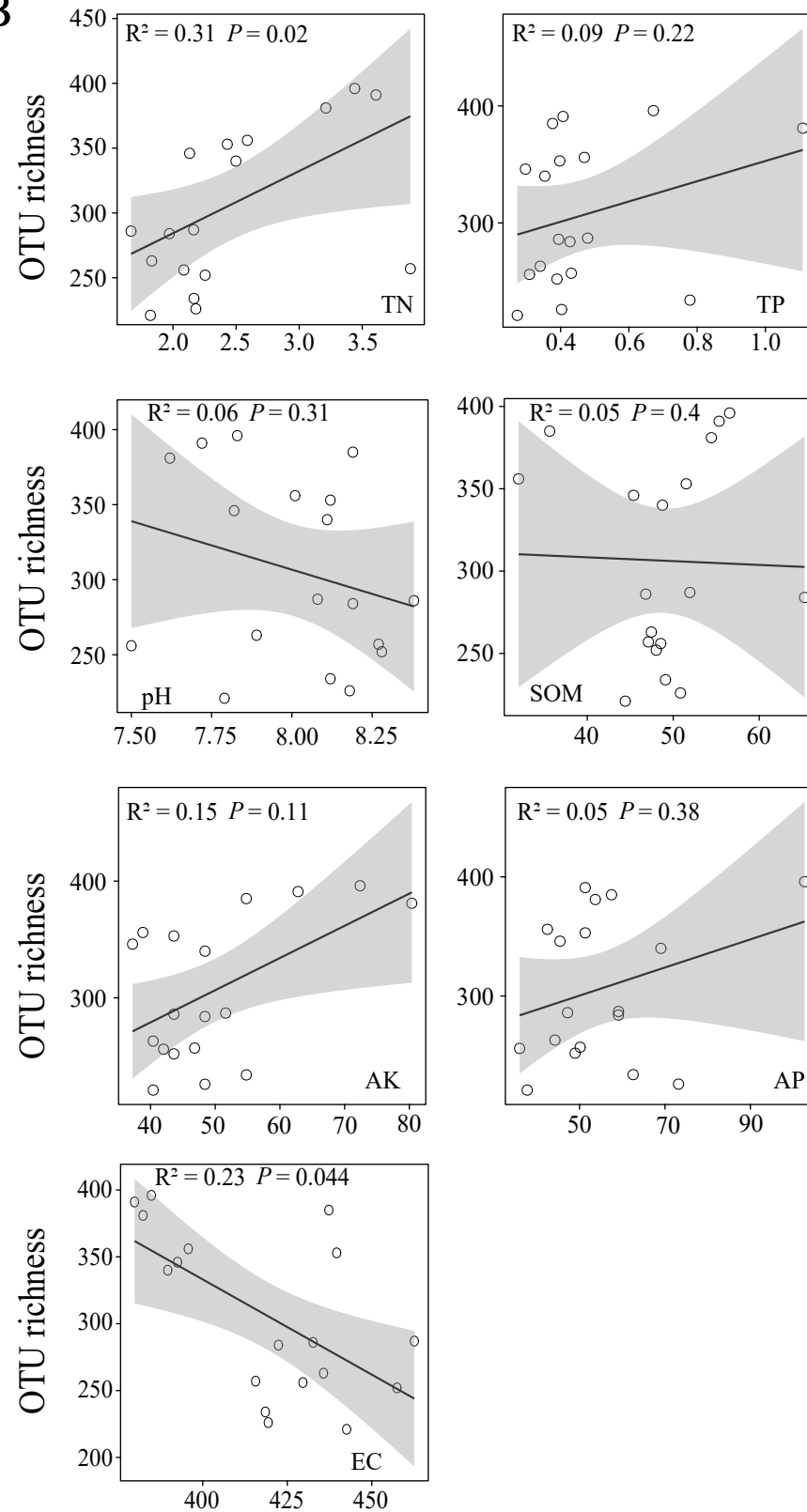

Supplement: Supplementary file 5 [file Image_2.pdf]

A

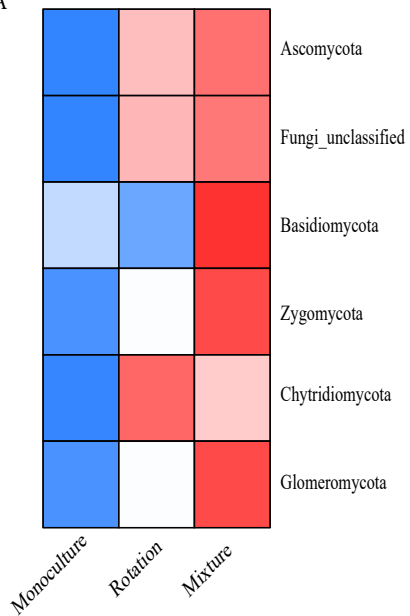

B

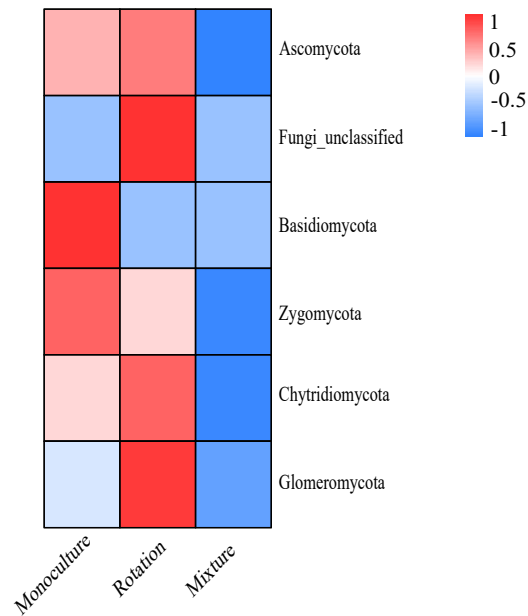

Supplement: Supplementary file 6 [file Image_3.pdf]

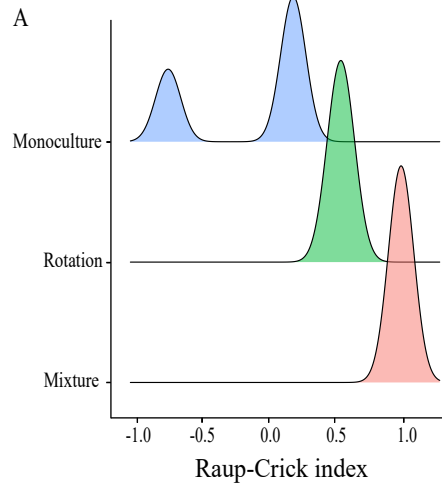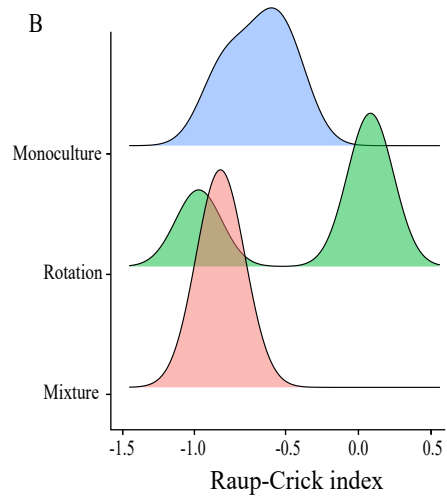

Supplement: Supplementary file 7 [file Image_4.pdf]

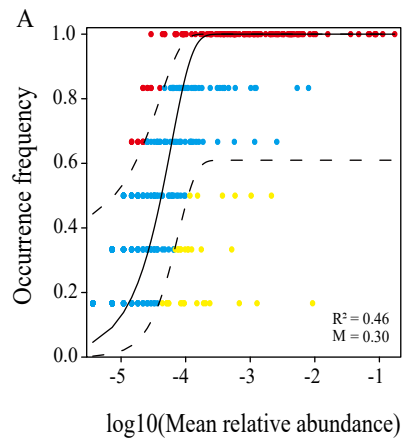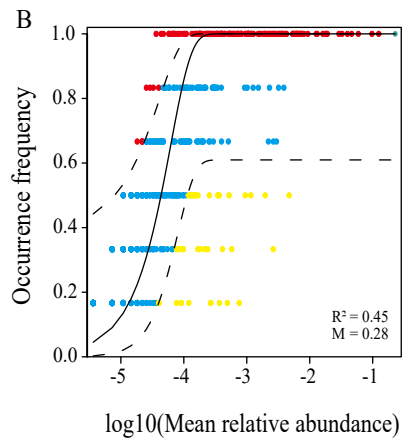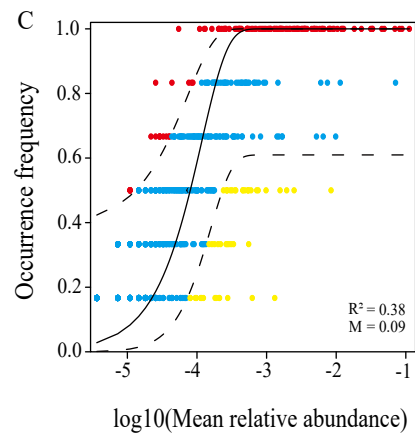

Supplement: Supplementary file 8 [file Image_5.pdf]

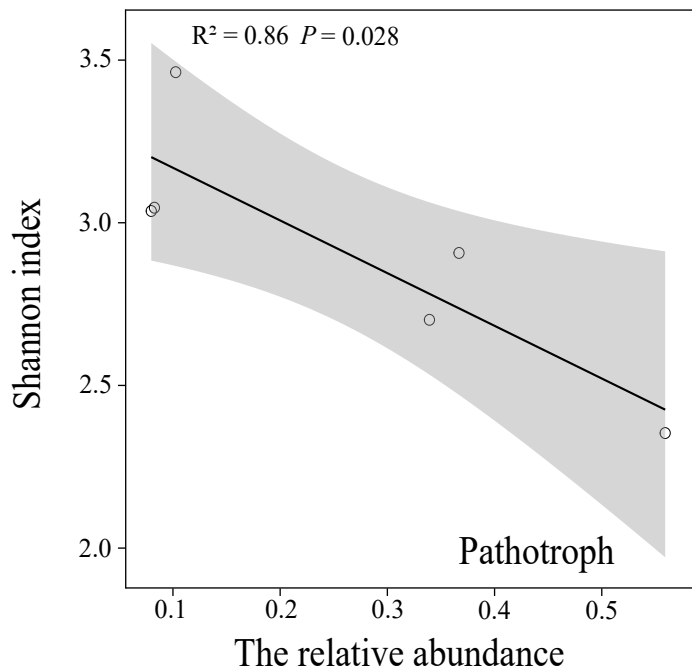

Supplement: Supplementary file 9 [file Image_6.pdf]
